# Molecular determinants of selective dopaminergic vulnerability in Parkinson’s disease: an update

**DOI:** 10.3389/fnana.2014.00152

**Published:** 2014-12-15

**Authors:** Lars Brichta, Paul Greengard

**Affiliations:** Laboratory of Molecular and Cellular Neuroscience, The Rockefeller UniversityNew York, NY, USA

**Keywords:** Parkinson’s disease, substantia nigra, ventral tegmental area, dopamine, selective vulnerability, differential vulnerability

## Abstract

Numerous disorders of the central nervous system (CNS) are attributed to the selective death of distinct neuronal cell populations. Interestingly, in many of these conditions, a specific subset of neurons is extremely prone to degeneration while other, very similar neurons are less affected or even spared for many years. In Parkinson’s disease (PD), the motor manifestations are primarily linked to the selective, progressive loss of dopaminergic (DA) neurons in the substantia nigra pars compacta (SNpc). In contrast, the very similar DA neurons in the ventral tegmental area (VTA) demonstrate a much lower degree of degeneration. Elucidating the molecular mechanisms underlying the phenomenon of differential DA vulnerability in PD has proven extremely challenging. Moreover, an increasing number of studies demonstrate that considerable molecular and electrophysiologic heterogeneity exists among the DA neurons within the SNpc as well as those within the VTA, adding yet another layer of complexity to the selective DA vulnerability observed in PD. The discovery of key pathways that regulate this differential susceptibility of DA neurons to degeneration holds great potential for the discovery of novel drug targets and the development of promising neuroprotective treatment strategies. This review provides an update on the molecular basis of the differential vulnerability of midbrain DA neurons in PD and highlights the most recent developments in this field.

## Introduction

The selective death of distinct neuronal cell populations is the key feature of many disorders of the central nervous system (CNS). The identity of the affected neurons as well as the pattern of neuronal degeneration are specific to the respective disorder and determine the clinical signs that are typically associated with the condition. Examples of diseases that are characterized by the selective degeneration of neurons in the CNS include Alzheimer’s disease, Parkinson’s disease (PD), Amyotrophic Lateral Sclerosis (ALS) and autosomal recessive proximal Spinal Muscular Atrophy. It is largely unknown why different groups of neurons are highly vulnerable to degeneration in different diseases. Moreover, it is unclear why, in a particular condition, some neurons are extremely prone to degeneration while other, very similar neurons are spared over years. In early Alzheimer’s disease, distinct subgroups of neurons in layer II of the entorhinal cortex, the subiculum, and the CA1 region of the hippocampus are particularly vulnerable to degeneration, while many other cortical and hippocampal regions do not show pathological signs at this disease stage (Morrison and Hof, 2002, 2007; Stranahan and Mattson, 2010). Amyotrophic Lateral Sclerosis affects both upper and lower motor neurons (Boillée et al., 2006; Talbot, 2014). However, cortical, spinal and lower cranial nerve motor neurons undergo degeneration early in the disease, while the motor neurons of Onuf’s nucleus, as well as the oculomotor, trochlear and abducens nerves remain largely unaffected from cell loss even at late disease stages (Alexianu et al., 1994; Kihira et al., 1997; von Lewinski and Keller, 2005). Autosomal recessive proximal spinal muscular atrophy is characterized by the progressive selective loss in particular of the lower motor neurons in the anterior horns of the spinal cord, while upper motor neurons are spared (Talbot and Davies, 2001). In PD, the motor manifestations are primarily linked to the selective loss of dopaminergic (DA) neurons in the substantia nigra pars compacta (SNpc; Brichta et al., 2013). In contrast, the very similar DA neurons in the ventral tegmental area (VTA) demonstrate a higher degree of resistance to degeneration (Dauer and Przedborski, 2003). Our current knowledge about the determinants of the differential vulnerability of SNpc and VTA DA neurons in PD is still extremely limited. Undoubtedly, discovery of the factors that mediate this differential vulnerability would have far-reaching implications for the development of promising neuroprotective strategies and the treatment of PD. This review provides an update on the molecular basis of the differential vulnerability of midbrain DA neurons in PD. We will focus on the most recent developments in this field and discuss these data in the light of previous findings and also regarding their relevance in explaining different aspects of the pattern of DA cell loss in PD.

## Midbrain DA neurons and their differential vulnerability in PD

**Figure 1:**
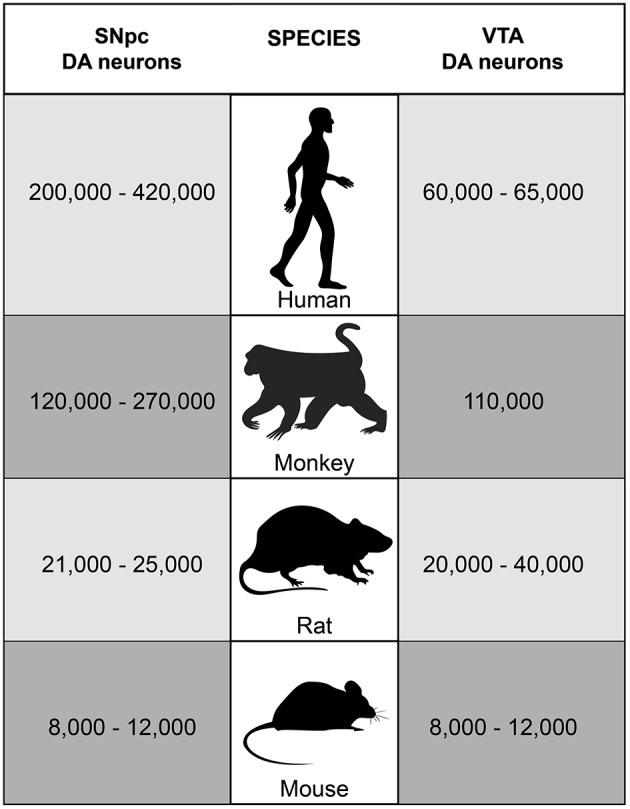
**Number of SNpc and VTA DA neurons in humans, monkeys, rats and mice**. In adult C57BL/6 mice, approximately 8,000–12,000 TH-positive neurons are located in each of the two neuron groups bilaterally (German et al., 1996; Nelson et al., 1996; Jackson-Lewis and Przedborski, 2007). Studies in rats revealed about 21,000–25,000 TH-positive neurons in the SNpc and about 20,000–40,000 TH-positive neurons in the VTA bilaterally (German and Manaye, 1993; Nair-Roberts et al., 2008). In monkeys and humans, much higher numbers of DA neurons are found in the midbrain. Stereology studies counted approximately 120,000–270,000 TH-positive neurons bilaterally in the SNpc and about 110,000 TH-positive neurons bilaterally in the VTA in rhesus and squirrel monkeys (Emborg et al., 1998; McCormack et al., 2004; Collier et al., 2007; Kanaan et al., 2007). In humans, about 200,000–420,000 TH-positive neurons bilaterally were reported for the SNpc in adults and about 60,000–65,000 TH-positive neurons for the VTA (Hirsch et al., 1988; McRitchie et al., 1997; Damier et al., 1999a; Chu et al., 2002).

The largest groups of DA neurons in the midbrain are located in very close proximity to each other in the SNpc (A9 group) and in the VTA (A10 group). SNpc and VTA DA neurons represent two of the nine major DA neuron groups in the mammalian brain as identified by staining for tyrosine hydroxylase (TH), the enzyme that catalyzes the rate-limiting step in the synthesis of dopamine (Björklund and Dunnett, 2007). In mice and rats, considerably fewer DA neurons are found in the midbrain than in monkeys and humans (Figure 1). Moreover, while SNpc and VTA contain comparable numbers of DA neurons in mice and rats, SNpc DA neurons outnumber VTA DA neurons in monkeys and humans. The variations among the reported counts of TH-positive neurons in the SNpc and the VTA for a specific species may at least in part be due to interindividual differences between study subjects, but are most likely the result of different statistical methods that were applied to calculate the total number of DA neurons in the two midbrain nuclei. In addition to the DA neurons, both SNpc and VTA also contain a considerable number of GABAergic neurons, while only a very few glutamatergic neurons are present whose location is mainly restricted to the VTA (Nair-Roberts et al., 2008).

SNpc DA neurons are heavily involved in the control of movement, whereas VTA DA neurons are responsible for the regulation of reward, emotional behavior and addiction. Both groups of neurons are characterized by distinct but overlapping projection patterns that have been extensively investigated in numerous tracing studies (comprehensively reviewed in (Bentivoglio and Morelli, 2005)). The majority of projections that originate from SNpc DA neurons innervate the dorsal striatum, and only some nigral fibers project to the ventral striatum and the cortex. In contrast, VTA DA neurons mainly project to the ventral striatum as well as cortical areas, while significantly fewer projections innervate the dorsal striatum. Both SNpc and VTA DA neurons send minor projections to additional brain regions including the globus pallidus, the subthalamic nucleus, and the habenula. Moreover, a minority of DA projections originating in the VTA projects to the amygdala. DA neurons located in the midbrain also sparsely innervate distinct hippocampal regions. The density of the DA innervation of a distinct brain region often varies significantly between different species. Interestingly, anatomic differences in the DA projection sites correlate with several dissimilarities observed in DA neuron function. A combination of retrograde tracing, electrophysiological and basic molecular studies in mice demonstrated that distinct subgroups of VTA and SNpc DA neurons in confined midbrain territories project to specific striatal, cortical and limbic target regions, and these neurons can be distinguished by their expression levels of dopamine transporter (DAT), their electrophysiological properties and their capacities for dopamine D_2_ autoreceptor signaling (Lammel et al., 2008).

Despite the fact that SNpc and VTA DA neurons generate, store and release the same neurotransmitter, that their cell bodies in the midbrain are localized in close proximity to each other, and that significant overlap exists in the brain areas that are innervated by the projections of these neurons, SNpc and VTA DA neurons exhibit a different susceptibility to degeneration in PD. The DA neurons in the SNpc are highly vulnerable to the fatal molecular mechanisms associated with the disease. Along with the formation of alpha-synuclein-rich intraneuronal protein aggregates termed Lewy Bodies in various brain regions (Spillantini et al., 1997; Braak et al., 2003; Burke et al., 2008), the progressive loss of pigmented SNpc DA neurons is a pathological hallmark of PD (Brichta et al., 2013). Calculations of the number of DA neurons (as identified by the expression of TH) in post-mortem tissue from patients with advanced idiopathic (non-genetic) PD and control subjects revealed that, on average, almost 80% of all SNpc DA neurons undergo degeneration in PD (Hirsch et al., 1988; Damier et al., 1999b). DA cell loss is most severe in the ventrolateral part of the SNpc (Halliday et al., 1996; Damier et al., 1999b; Braak et al., 2003), which was also unequivocally demonstrated in a more current study that investigated a large set of post-mortem brain samples obtained from patients with PD (Kordower et al., 2013). These data show that DA cell loss in the SNpc follows a specific pattern and suggest that subtle molecular differences exist among subgroups of SNpc DA neurons. In contrast to SNpc DA neurons, the very similar DA neurons in the VTA are more resistant to degeneration. In their studies, Damier et al. and Hirsch et al. analyzed the number of both SNpc and VTA DA neurons, providing cell counts that can be directly compared with each other due to the investigation of the same midbrain tissue samples and the application of the same statistical methods. The average loss of VTA DA neurons observed in patients with advanced idiopathic PD as compared to unaffected controls was estimated to be only about 50% which is a much lower percentage of DA neuron loss than in the SNpc (Hirsch et al., 1988; Damier et al., 1999b). These data strongly suggest that specific modifiers partially protect VTA DA neurons from degeneration as compared to SNpc DA neurons, and/or that specific modifiers increase the vulnerability of SNpc DA neurons to PD-associated cell loss as compared to VTA DA neurons.

PD can be caused by non-genetic or genetic factors (Cookson and Bandmann, 2010; Houlden and Singleton, 2012; Hirsch et al., 2013; Singleton et al., 2013). However, differential vulnerability among midbrain DA neurons is observed regardless of the disease etiology. Neuropathological findings in published cases with various genetic forms of PD include loss of SNpc DA neurons without describing VTA DA neuron degeneration (Mori et al., 1998; Spira et al., 2001; Gouider-Khouja et al., 2003; Farrer et al., 2004; Zarranz et al., 2004; Zimprich et al., 2004; Pramstaller et al., 2005; Hasegawa et al., 2009). Thus, it is reasonable to conclude that differential DA neuron vulnerability does not depend on the factor triggering PD, but is based on slightly varying intrinsic properties of subgroups of these neurons.

## Comparative expression profiling of SNpc and VTA DA neurons

A highly promising approach to identify intrinsic factors that distinguish SNpc from VTA DA neurons and that mediate the differential vulnerability of these neurons to degeneration in PD is the comparative expression profiling of SNpc and VTA DA neurons. Technologies such as laser-capture microdissection (LCM), microarray and next-generation sequencing facilitate the isolation of specific types of DA neurons and the characterization of their gene expression profiles. Comparative expression profiling studies in human brain samples are surprisingly rare. An early study in post-mortem tissue samples from control subjects described the isolation of DA neurons from several different brain regions including the SNpc and the VTA and the subsequent generation of cell-specific RNA expression profiles using a fingerprinting approach (Lu et al., 2004). However, this investigation did not go beyond the description of a methodological protocol and did not provide a list of differentially expressed transcripts or any functional analyses. Since then, several studies have focused on the analysis of the gene expression profiles of laser-captured SNpc DA neurons in patients with PD and control subjects, but a comparison between SNpc and VTA DA neurons was not pursued (Cantuti-Castelvetri et al., 2007; Simunovic et al., 2009; Zheng et al., 2010). The lack of such comparative expression profiling studies may be due to the fact that many brain banks have difficulties providing tissue samples containing VTA DA neurons. It is often standard protocol that donated brains are crudely divided into the left and right hemispheres which are then processed separately. Since VTA DA neurons are located along or close to the midline, the tissue containing these neurons is often destroyed.

In contrast to the lack of human studies, three different studies have applied LCM in combination with microarray analysis to compare the expression profiles of SNpc and VTA DA neurons in rodents (Grimm et al., 2004; Chung et al., 2005; Greene et al., 2005). Another comparative study was carried out by manually dissecting whole SNpc and VTA followed by serial analysis of gene expression (SAGE). However, the main goal of this analysis was not the elucidation of transcripts that are differentially expressed between SNpc and VTA, but rather the overall comparison of the gene expression patterns of 11 different mouse brain regions (Brochier et al., 2008). The rationale for these investigations was that both SNpc and VTA are conserved between humans, mice and rats. Moreover, the differential susceptibility of these two DA neuron groups to degeneration in PD can be reproduced with the neurotoxin MPTP in various animal models for PD (Seniuk et al., 1990; Sirinathsinghji et al., 1992; Muthane et al., 1994; Varastet et al., 1994; Jackson-Lewis et al., 1995). All three comparative microarray analyses demonstrated that SNpc and VTA DA neurons are closely related and only <1% (Grimm et al., 2004) or <3% (Greene et al., 2005) of all detected genes were differentially expressed. A functional analysis of the gene expression patterns of SNpc and VTA DA neurons suggested that many transcripts related to metabolism, transcripts encoding mitochondrial proteins, transcripts related to lipid, protein and vesicle-mediated transport and transcripts related to kinase/phosphatase signaling are more highly expressed in SNpc neurons, whereas transcripts implicated in axon guidance or in neuropeptide signaling were mainly enriched in VTA neurons (Chung et al., 2005; Greene et al., 2005). Grimm et al. found that many genes encoding for proteins involved in synaptic plasticity, cell survival (neuroprotection, detoxification) or axonal pathfinding and neuronal migration are more highly expressed in the VTA (Grimm et al., 2004). Several of the differentially expressed gene candidates were confirmed by all three microarray studies and a small selection of interesting candidates was discussed elsewhere (Greene, 2006). However, the overall overlap observed between these three studies is limited which, at least in part, might be due to the facts that two studies were carried out in rats and one study in mice, and that different microarray chips with comparably small numbers of probe sets were used for sample analysis. Consequently, even with these expression data at hand, the identification of specific key molecular pathways that clearly mediate the selective DA neuron vulnerability in PD has remained challenging. Chung et al. investigated two VTA DA neuron-enriched transcripts in more detail: G-substrate and Rab3b (Chung et al., 2007, 2009). G-substrate is an endogenous inhibitor of Serine/Threonine protein phosphatases, while Rab3b is an isoform belonging to the Rab3 GTPase protein family that is enriched in synaptic vesicles in neurons and involved in synaptic vesicle trafficking. Interestingly, the virus-mediated overexpression of either candidate in SNpc DA neurons in rats protected these neurons from 6-OHDA-mediated neurodegeneration. However, protein overexpression in a different animal model that more closely resembles the slow, progressive DA neuron loss in PD, such as the subacute MPTP-induced mouse model, was not pursued. Moreover, convincing data indicating that boosting G-substrate or Rab3b function may attenuate SNpc DA neuron degeneration in PD are lacking thus far. Additionally, the development of small molecules that are able to specifically activate Rab3b or G-substrate in the SNpc might be hampered by the expression of both proteins in brain regions other than the midbrain and their apparent involvement in various different signaling pathways whose manipulation could potentially interfere with normal brain function (Endo et al., 2009; Tsetsenis et al., 2011).

Surprisingly, the elucidation of the molecular determinants of the differential vulnerability of SNpc and VTA DA neurons in PD has received little attention since. One study investigated the transcriptional changes in SNpc and VTA DA neurons in mice after the injection of saline or 4 mg MPTP/kg/day for two days or 10 days (Phani et al., 2010). This injection schedule is unconventional and has not been described in the literature. The authors observed a 30% loss of SNpc DA neurons in MPTP-treated animals after two days and a 70% loss after 10 days. In the VTA, only 10% of DA neurons degenerated and this loss did not progress over time. Mice were sacrificed seven days after the final injection. Following the isolation of DA neurons by LCM and RNA purification, expression analysis was carried out using microarrays. The conclusions of this investigation remained very general, stating that a number of genes were up-regulated in the VTA as compared to the SNpc after exposure to MPTP. Almost no overlap existed between the genes up-regulated before and after MPTP treatment, and only very few genes were consistently up-regulated at two days and at 10 days after MPTP treatment, which is somewhat in contrast to the fact that no progressive VTA DA neuron loss was observed. These results suggest that the expression signature of VTA DA neurons may be very variable depending on the respective experimental conditions. However, independent confirmation of this finding using the same or a similar animal model is desired. In a follow-up study, Phani et al. suggested that the exogenous addition of the peptide gremlin, whose mRNA levels were elevated in the VTA DA neurons after MPTP treatment of mice, protects SNpc DA neurons from MPP^+^-induced degeneration, possibly via vascular endothelial growth factor receptor 2 (Phani et al., 2013). Data from additional animal models or human data were not presented, and due to its peptide nature, the delivery of gremlin to the SNpc could be problematic for the development of a therapy. Another study laser-captured DA neurons from rats infused with rotenone for 28 days or from mice acutely treated with MPTP and investigated the levels of a set of transcripts related to energy metabolism using quantitative real-time PCR (Greene et al., 2010). SNpc DA neurons demonstrated higher baseline levels and more pronounced changes in the levels of these transcripts than VTA DA neurons. These findings are in agreement with those from Chung et al. and are interesting particularly in light of the predicted higher metabolic demand of SNpc DA neurons which is discussed below. The mechanisms underlying these DA neuron subtype-specific observations remain to be elucidated.

Future studies addressing the gene expression differences between SNpc and VTA DA neurons would be of great interest, in particular if next-generation sequencing is used as an unbiased method to unequivocally identify and more accurately quantify the entirety of all transcripts that are expressed at different stages of DA neuron degeneration. In addition, the preferred application of the more sophisticated bacTRAP methodology over LCM would most likely result in a significant refinement of the available data sets that describe the gene expression profiles of midbrain DA neurons, as bacTRAP focuses on the specific analysis of translated messages and does not include translationally silent RNAs (Doyle et al., 2008; Heiman et al., 2008).

## G-protein-activated inwardly rectifying potassium channel 2 (GIRK2)

GIRK2 has long been considered a protein whose expression pattern may potentially correlate with the differential vulnerability of subgroups of midbrain DA neurons. It represents one family member of a group of ion channels and is involved in the regulation of neuronal activity (Kobayashi and Ikeda, 2006). Weaver mice carry a homozygous missense mutation in the gene encoding for GIRK2 (Patil et al., 1995) which leads to the progressive loss of SNpc DA neurons that starts after postnatal day 7 and reaches a 69% loss on postnatal day 90 (Triarhou et al., 1988; Verney et al., 1995). Ventral SNpc DA neurons are most severely affected by the degeneration (Graybiel et al., 1990). In contrast, the loss of DA neurons in the VTA is delayed and less severe than in the SNpc (Triarhou et al., 1988; Graybiel et al., 1990; Martí et al., 2000, 2007). Although the conclusions presented in these studies are convincing, it is noteworthy that these investigations did not apply state-of-the-art stereological methods to determine the number of surviving DA neurons. Therefore, the presented percentages and cell numbers may slightly deviate from the actual values. Also, caution is required when interpreting these findings in the context of PD, as degeneration in weaver mice starts before the complete maturation of SNpc DA neurons, which may introduce a developmental component that is not present in PD.

Initial *in-situ* hybridization and immunohistochemistry studies in adult control mice showed that most SNpc DA neurons and lateral VTA DA neurons express *Girk2*/GIRK2, while the majority of medial VTA DA neurons seemed to lack detectable *Girk2*/GIRK2 levels (Schein et al., 1998). Schein et al. also suggested that the fraction of VTA DA neurons that are *Girk2*/GIRK2-positive express lower levels than *Girk2*/GIRK2-positive SNpc DA neurons (Schein et al., 1998). These findings implied an overall enrichment of *Girk2*/GIRK2 in the SNpc as compared to the VTA, although the absence or presence of *Girk2*/GIRK2 could not serve as a qualitative marker to clearly distinguish between these two DA nuclei.

Several subsequent studies in humans, mice and rats then suggested that the expression of GIRK2 distinguishes between the DA neurons in the dorsal and ventral tier of the SNpc, as GIRK2 was detected particularly in ventral tier SNpc DA neurons (Mendez et al., 2005; Thompson et al., 2005; Björklund and Dunnett, 2007). These data gave rise to speculations that GIRK2 may not only differentiate between SNpc and VTA DA neurons, but it may also serve as a molecular marker for ventral SNpc DA neurons which show the highest vulnerability in PD. However, a functional explanation for this observation was not provided.

In an extensive immunohistochemistry study in tissue samples from five human brain donors and six wild-type mice, Reyes et al. recently revisited the expression of GIRK2 protein in midbrain DA neurons (Reyes et al., 2012). This study revealed that some level of GIRK2 is expressed in almost all TH-positive neurons in the SNpc and the VTA in mice and in almost all neuromelanin-positive neurons in human midbrain. In both species, most GIRK2-positive SNpc DA neurons showed strong expression of GIRK2, while few SNpc DA neurons were characterized by weak GIRK2 expression. Importantly, no significant difference was observed between the GIRK2 expression pattern in dorsal and ventral tier SNpc DA neurons. In both tiers, the vast majority of SNpc DA neurons were positive for GIRK2, and similarly high numbers of these neurons showed high GIRK2 expression levels. These findings suggest that GIRK2 expression does not distinguish between dorsal and ventral tier SNpc DA neurons. Most VTA TH-positive neurons in mice and humans also expressed some level of GIRK2. Therefore, the qualitative assessment of GIRK2 expression cannot serve as a tool to discriminate between SNpc and VTA DA neurons. However, strong GIRK2 expression was detected in about half of the VTA DA neurons, which represents a large fraction of VTA DA neurons but is a considerably smaller number than that of DA neurons with high GIRK2 levels in the SNpc. Consistent with these data, Chung et al. recently reported that, overall,* Girk2* mRNA levels are higher in SNpc DA neurons than in VTA DA neurons (Chung et al., 2005). This was demonstrated in adult wild-type mice using LCM of DA neurons followed by quantitative real-time PCR.

Strikingly, another recent immunohistochemistry study in mice further confirmed that GIRK2 is expressed in almost all SNpc TH-positive neurons in both ventral and dorsal tiers, as well as the majority of VTA TH-positive neurons (Fu et al., 2012). Similar results were obtained in an immunohistochemistry study in rats (Eulitz et al., 2007). Fu et al. did not confirm that more SNpc DA neurons express high levels of GIRK2 than VTA DA neurons (Fu et al., 2012).

Taken together, these results indicate that GIRK2 is unlikely to be a marker to distinguish between dorsal and ventral tier SNpc DA neurons, and that the GIRK2 expression pattern does not correlate with the differential vulnerability of these two SNpc DA neuron subgroups in PD. It is unclear at this point if GIRK2 contributes to the selective vulnerability of SNpc and VTA DA neurons. It appears that a larger fraction of SNpc DA neurons than VTA DA neurons express high GIRK2 levels, which is consistent with the overall enrichment of *Girk2* transcript in the SNpc as compared to the VTA. However, the observations that the majority of VTA DA neurons do express some GIRK2 and that a considerable percentage of VTA DA neurons contain GIRK2 levels as high as most SNpc DA neurons complicate matters and weaken the potential relevance of GIRK2 as a therapeutic target, as these findings do not unequivocally correlate with the differential susceptibility of SNpc and VTA DA neurons in PD.

## Calbindin

Calbindin is a calcium-binding protein that is widely expressed in many brain areas and involved in the regulation of intracellular calcium levels (Liu and Graybiel, 1992). Similar to GIRK2, many studies have suggested calbindin as a marker to distinguish between midbrain DA neurons with different susceptibility to degeneration in PD. Gene expression profiling studies in rats and mice applying LCM in combination with microarray analysis demonstrated that calbindin transcripts are enriched in VTA DA neurons as compared to SNpc DA neurons (Chung et al., 2005; Greene et al., 2005). Immunostaining in mice and rats revealed that TH-positive cells in both SNpc and VTA express calbindin. However, the number of TH-positive VTA neurons that co-express calbindin is higher than that of TH-positive SNpc neurons (Liang et al., 1996; Björklund and Dunnett, 2007). Within the SNpc, TH-positive, calbindin-expressing neurons were mainly found in the dorsal tier. A similar calbindin expression pattern was observed in human midbrain (Mendez et al., 2005).

In their recent immunohistochemistry study in tissue samples derived from five human controls and six wild-type mice, Reyes et al. confirmed many of the previously reported data regarding the expression of calbindin in midbrain DA neurons (Reyes et al., 2012). In the mouse and human SNpc, fewer TH- or neuromelanin-positive neurons contained calbindin than in the VTA. These neurons were mainly localized in the medial and lateral SNpc, and a very small number of calbindin-positive DA neurons were also detected in the dorsal SNpc. However, calbindin-positive neurons were completely absent from the ventral tier of the SNpc. A much larger fraction of TH- or neuromelanin-positive VTA neurons in mice and humans, respectively, contained calbindin. These findings correlate with the overall enrichment of calbindin transcripts in VTA DA neurons over SNpc DA neurons that was reported in the LCM/microarray expression studies discussed above (Chung et al., 2005; Greene et al., 2005). Nevertheless, the expression pattern of calbindin does not accurately reflect the different degrees of DA neuron vulnerability. Many but not all VTA DA neurons express calbindin, and some SNpc DA neurons express calbindin as well, such that calbindin expression cannot serve as a qualitative marker to clearly distinguish between SNpc and VTA. Moreover, calbindin is indeed absent from ventral tier SNpc DA neurons, but not all dorsal tier SNpc DA neurons contain calbindin, which raises the question how closely the expression pattern of calbindin correlates with the differential vulnerability of dorsal and ventral tier SNpc DA neurons.

Fu et al. also revisited the expression of calbindin in mouse midbrain using immunohistochemistry and confirmed that no SNpc DA neurons in the ventral tier express calbindin (Fu et al., 2012). In the dorsal tier, a signal for calbindin was detected in fewer than 2% of SNpc DA neurons, suggesting that some difference exists between the ventral and dorsal tiers of the SNpc. However, many dorsal tier SNpc DA neurons do not follow this expression pattern as they are calbindin-negative, underscoring the view that calbindin does not represent a suitable marker for different degrees of DA neuron vulnerability. Also in agreement with previously published data, it was demonstrated that some TH-positive neurons outside the dorsal tier of the SNpc express calbindin, particularly in the medial and lateral SNpc, but the number of TH- and calbindin-positive neurons in these SNpc regions was lower than that in the VTA.

Several years ago, it was demonstrated that the presence or absence of calbindin in SNpc and VTA DA neurons correlates with distinct electrophysiological features of these DA neuron subgroups that are mediated by hyperpolarization-activated, cyclic nucleotide-regulated cation (HCN) channels (Neuhoff et al., 2002). Interestingly, another electrophysiological study now identified a novel function for calbindin in midbrain DA neurons expressing this protein (Pan and Ryan, 2012). Using rat midbrain DA neuronal cultures, an inverse correlation was demonstrated between the expression level of calbindin and the probability of vesicle exocytosis, suggesting that calbindin is involved in the control of dopamine release. Based on these results, it appears that calbindin-negative SNpc DA neurons are characterized by a higher dopamine release probability than most VTA DA neurons which are calbindin-positive. However, this study did not address the heterogeneous expression of calbindin that is observed among SNpc DA neurons and also among VTA DA neurons. No experiments were carried out to investigate if and how this mechanism can be linked to the differential vulnerability of DA neurons in PD. Early investigations indicated that calbindin does not directly mediate protection, as the same subgroups of DA neurons were spared from degeneration in MPTP-treated calbindin knockout mice and in their wild-type littermates (Airaksinen et al., 1997). However, some caution is recommended when interpreting these data, as this study did not yet apply the stereological methods that are currently considered state-of-the-art to accurately determine neuronal numbers. In consideration of the available data, and based on the complex expression pattern of calbindin in SNpc and VTA DA neurons, many questions remain regarding the suitability of calbindin as a marker for DA neurons with increased resistance to degeneration in PD and the potential role of calbindin in mediating differential DA vulnerability.

## Receptors and transporters

DA neurons express a variety of cell surface receptors and transporters that may serve as potential binding or entry sites for neurotoxic substances. Providing proof of concept for this hypothesis, it is well established that MPP^+^ is taken up specifically by DA neurons via DAT, resulting in mitochondrial damage and ultimately DA neurodegeneration (Dauer and Przedborski, 2003). In the context of differential DA vulnerability, it may be feasible to speculate that in particular the most vulnerable DA neurons express a unique set of transporters and/or receptors that facilitate the uptake of or transmit extracellular signals initiated by PD-related neurotoxins. A number of studies have focused on the expression pattern of various receptors in the midbrain DA system over the years. In one of the most recent investigations, Reyes et al. applied immunohistochemistry and co-staining for TH to analyze the expression patterns of the five dopamine receptors and DAT in post-mortem human midbrain tissue in great detail (Reyes et al., 2013a). The dopamine receptors D1, D2, D3 and D5 were detected in most SNpc and VTA DA neurons, although slightly fewer VTA DA neurons were positive for D1 and D2, respectively, than SNpc DA neurons. Notably, considerably more SNpc DA neurons than VTA DA neurons showed high expression levels of the D1 or D2 receptor. Similarly, while most midbrain DA neurons expressed DAT, significantly more SNpc DA neurons than VTA DA neurons expressed high levels of DAT. Reyes et al. also investigated the levels of glycosylated DAT which is the mature, highly functional version of this transporter (Li et al., 2004). While more than 90% of the analyzed SNpc DA neurons and more than 80% of the analyzed VTA DA neurons expressed some level of glycosylated DAT, high levels of glycosylated DAT were found, in particular, in a fraction of DA neurons located in the most vulnerable ventral tier of the SNpc. This is in agreement with the findings of another immunohistochemistry study in rats and human midbrain (Afonso-Oramas et al., 2009). However, ventral tier SNpc DA neurons did not show uniform expression levels of glycosylated DAT, and fewer than 20% of these neurons were characterized by high levels. If the reported differences in the expression levels of some dopamine receptors and DAT indeed contribute to the differential DA vulnerability, the underlying mechanism remains to be determined. Importantly, D1, D2 and glycosylated DAT are present in almost all midbrain DA neurons, only at slightly different levels, and it is unclear how these rather small expression differences would be able to account for the varying degrees of vulnerability observed between SNpc and VTA DA neurons or between DA neurons in the dorsal and ventral tiers of the SNpc.

In this context, it is noteworthy to mention that SNpc and VTA DA neurons receive input from a considerable number of different brain areas. In an elegant study that was carried out in mice, the Cre/LoxP system was combined with rabies virus-based transsynaptic retrograde tracing to generate a comprehensive list of the brain areas that provide monosynaptic input to SNpc or VTA DA neurons or both (Watabe-Uchida et al., 2012). Not surprisingly, it was demonstrated that each group of midbrain DA neurons is innervated by an overlapping, yet distinct set of brain areas. These data clearly show that SNpc and VTA DA neurons are wired differently in the brain and that each of these two DA neuron groups receives and integrates a distinct set of signals. Although not all of the findings from mice may carry over to the human brain, it can be speculated that SNpc DA neurons may receive toxic inputs that do not reach the VTA, or VTA DA neurons may receive protective signals that do not reach the SNpc. Together, the slightly different connectivity of SNpc and VTA DA neurons within the brain and small differences in the expression levels of specific receptors at the cell surface of SNpc and VTA DA neurons may contribute to the differential vulnerability of these neurons observed in PD. This hypothesis is also interesting in light of a recent investigation in mice which showed that the introduction of preformed fibrils of alpha-synuclein to a particular brain region led to the progressive propagation of Lewy Body/Lewy neurite pathology in other brain regions based on the connectivity of neurons and cell-to-cell transmission (Luk et al., 2012). After the stereotaxic injection of preformed fibrils consisting of recombinant mouse alpha-synuclein into the dorsal striatum, severe alpha-synuclein pathology was observed in the SNpc TH-positive neurons but not in the VTA TH-positive neurons. This is consistent with the fact that the dorsal striatum is heavily innervated by the SNpc DA neurons while VTA DA neurons send significantly fewer projections to this area, providing an example of how differences in the neuronal connectivity of midbrain DA neurons can lead to the selective damage of distinct DA neuron groups. Importantly, the propagation of alpha-synuclein pathology is a process that very likely also occurs in the human brain, which has been demonstrated by the identification of alpha-synuclein-positive inclusions in DA neuron grafts years after transplantation into the striatum of patients with PD (Kordower et al., 2008a,b; Li et al., 2008).

## Electrophysiological determinants

It has long been suggested that SNpc DA neurons are characterized by autonomous pacemaking which is driven by calcium channels (Nedergaard et al., 1993; Mercuri et al., 1994). However, one of the major findings regarding the differential vulnerability of SNpc and VTA DA neurons came from a well-designed electrophysiological study in mice which demonstrated that only adult SNpc DA neurons rely on L-type Ca_v_1.3 calcium channels for pacemaking, whereas juvenile SNpc DA neurons and VTA DA neurons rely on voltage-dependent sodium channels (Chan et al., 2007). This reliance of adult SNpc DA neurons on calcium for pacemaking seems to result in increased cytosolic calcium levels and elevated levels of oxidative stress, which could explain the higher vulnerability of SNpc DA neurons to damaging insults and degeneration in PD (Guzman et al., 2010). Another study in cultured murine midbrain DA neurons demonstrated that the reliance of SNpc DA neurons on calcium for pacemaking facilitates the L-DOPA-induced increase of dopamine to cytotoxic levels (Mosharov et al., 2009). This L-DOPA-induced increase of intracellular dopamine levels was not observed in VTA DA neurons, which provided further insight into physiological differences between SNpc and VTA DA neurons. However, the molecular basis of these observations remains largely unclear. It is unknown which factors and pathways force maturing SNpc DA neurons into using calcium channels for pacemaking, and why and how VTA neurons escape the switch from sodium to calcium channels. Moreover, it is not understood which endogenous mechanisms SNpc DA neurons possess to counterbalance the continuous, calcium-related oxidative stress in order to survive under control conditions. Clearly, the identification of these SNpc-specific pro-survival mechanisms has the potential to lead to the development of exciting novel therapeutic targets for the treatment of PD.

Electrophysiological differences between SNpc and VTA DA neurons were also revealed in a study focusing on the role of Kir6.2 and SUR1 subunit-containing ATP-sensitive potassium (K-ATP) channels in DA neurodegeneration in mice (Liss et al., 2005). In mouse brain slice cultures treated with the mitochondrial neurotoxins MPP^+^ or rotenone, spontaneous electrical activity was abolished exclusively in SNpc DA neurons, while VTA DA neurons remained unaffected. This was explained by the selective activation of K-ATP channels in SNpc DA neurons. Since K-ATP channels in SNpc and VTA DA neurons consist of the same subunits, the molecular composition of the channels did not account for these findings. However, the study suggested that the different responses of SNpc and VTA DA neurons may be due to different degrees of mitochondrial uncoupling, which controls the activation of K-ATP channels, most likely via different levels of reactive oxygen species (ROS). These findings are interesting and warrant further investigation and confirmation of these mechanisms *in vivo*. Interestingly, using LCM and mRNA quantification, a subsequent investigation provided evidence for the increased expression of the K-ATP channel subunit *SUR1* in SNpc DA neurons from patients with PD as compared to controls (Schiemann et al., 2012). Moreover, based on intra-operative measurements in PD patients, it was suggested that K-ATP channel-dependent burst firing is elevated in surviving DA neurons in the SNpc. Although the latter findings in particular need to be interpreted with caution due to the obvious lack of suitable control measurements, these data may further argue for a role of K-ATP channels in the degeneration specifically of SNpc DA neurons.

Bishop et al. established a preliminary link between PD caused by PINK1 mutations, calcium, and the different electrophysiological properties of SNpc and VTA DA neurons (Bishop et al., 2010). A comparison of brain slices from wild-type and *Pink1* knockout mice revealed that PINK1 deficiency in SNpc DA neurons is associated with irregular firing patterns due to the reduced activation of small-conductance calcium-activated potassium channels which was caused by impaired calcium release from the mitochondria and the endoplasmic reticulum. The authors speculated that the observed changes in the SNpc DA neuron firing pattern may eventually contribute to the increased calcium burden of these neurons and further elevate cellular stress. However, direct evidence for this particular hypothesis was not provided in the study. In contrast, VTA DA neurons did not change their firing patterns as a result of PINK1 deficiency, although the activity of small-conductance calcium-activated potassium channels was similarly decreased as in SNpc DA neurons. This was explained by the fact that SK3, the small-conductance calcium-activated potassium channel member whose mRNA is most abundant in midbrain DA neurons, is expressed at lower levels in VTA than in SNpc DA neurons and does not control certain aspects of pacemaking in the VTA as it does in the SNpc (Wolfart et al., 2001). Taken together, these findings are certainly of interest and demonstrate that the different molecular profiles of SNpc and VTA DA neurons may have significant consequences for the physiologic properties of these neurons and their differential response to PD-related changes. However, some of the conclusions of this study are still preliminary and require further investigation to identify all of the molecular components involved in mediating the differential response of SNpc and VTA DA neurons. It would also be of interest to compare the effects of PINK1 deficiency with PINK1 overexpression or to investigate the consequences that other PD-related mutations may have on DA neuron electrophysiology.

## Developmental transcription factors

The developmental formation of the midbrain, the differentiation of DA progenitor cells, and the induction of DA neuron markers are complex processes that require the spatial and temporal expression of specific sets of transcription factors (reviewed in (Smidt et al., 2003)). It has been demonstrated in mice that a close correlation exists between the developmental position of different DA progenitors, the expression pattern of specific protein markers that are typically present in subsets of these cells, and the vulnerability of the corresponding adult DA neurons in PD (Smits et al., 2013). Interestingly, some of the transcription factors expressed during neurogenesis remain expressed in the DA neurons during adulthood, and based on their expression patterns in SNpc and VTA DA neurons, a role in mediating the differential vulnerability of midbrain DA neurons has been hypothesized for these proteins.

Pituitary homeobox 3 (PITX3) is a transcription factor that is crucial for the development of SNpc DA neurons. Aphakia mice carry a homozygous null mutation in the* Pitx3* locus which results in the lack of PITX3 expression in the midbrain (Semina et al., 2000; van den Munckhof et al., 2003). While midbrain DA neurons appear normal in aphakia mice until embryonal day E12.5, PITX3 deficiency then interferes with the further development and the maintainance of SNpc DA neurons, resulting in severe DA neuron loss (Hwang et al., 2003; Nunes et al., 2003; van den Munckhof et al., 2003). In contrast, VTA DA neurons were observed to first develop normally. However, using unbiased stereology to estimate the number of TH-positive neurons, significant loss of VTA DA neurons was found in adult aphakia mice at the age of ~3 months (van den Munckhof et al., 2003; Luk et al., 2013). Although this loss was less severe than in the SNpc, these data suggest that PITX3 may also play a role for the long-term survival of VTA DA neurons.

For wild-type mice, it was reported that PITX3 is mainly expressed in the DA neurons in the ventral SNpc and in about 50% of VTA DA neurons without demonstrating non-DA neuron expression in the midbrain (van den Munckhof et al., 2003). The DA neurons in the dorsal SNpc were mainly PITX3-negative, which is consistent with the finding that in aphakia mice, some TH-positive cells were spared in the dorsal tier of the SNpc as these neurons may not require PITX3 to survive (van den Munckhof et al., 2003). The expression pattern of PITX3 in the midbrain has been investigated in several studies since and the reported results are controversial. In a study in three- to four-week-old rats, co-immunofluorescence staining for TH and PITX3 revealed that almost all SNpc and VTA DA neurons express PITX3 (Korotkova et al., 2005). A qualitative difference in PITX3 expression between the dorsal and ventral tiers of the SNpc was not observed. Two additional, very recent studies focused on the expression pattern of PITX3 in the midbrain. Luk et al. confirmed the localization of PITX3-positive SNpc DA neurons predominantly to the ventral tier and of PITX3-negative SNpc DA neurons to the dorsal tier in wild-type mice (Luk et al., 2013). It was reported that, in the VTA, many TH-positive neurons contained PITX3, and PITX3-positive and PITX3-negative DA neurons appeared intermixed with each other. However, it is not entirely clear from this study how many VTA TH-positive neurons expressed PITX3. Furthermore, Luk et al. compared the expression pattern of PITX3 with that of calbindin and found that the majority of SNpc DA neurons expressed either PITX3 or calbindin, but not both. In agreement with this observation, it was demonstrated in aphakia mice that the PITX3-negative DA neurons in the dorsal tier of the SNpc which escape degeneration are positive for calbindin. In contrast, the expression of calbindin in PITX3-negative VTA DA neurons was not sufficient to prevent degeneration of all of these neurons, which could point to a potential SNpc-specific survival mechanism of DA neurons expressing calbindin while lacking PITX3. Unfortunately, these findings are currently limited to the description of expression patterns, and a causal relationship between the absence of PITX3, the presence of calbindin and increased resistance to degeneration was not provided. Importantly, the potential function of PITX3 in adult midbrain DA neurons and its relevance for DA neuron vulnerability remains unclear. Immunostaining of brain slices obtained from wild-type mice after treatment with MPTP suggested that PITX3-expressing DA neurons are more vulnerable to the effects of this neurotoxin than PITX3-negative SNpc neurons. While it is well known that ventral SNpc DA neurons are more vulnerable to degeneration than dorsal SNpc DA neurons, convincing evidence for a direct link between this observation and the expression of PITX3 is missing. Interestingly, heterozygous aphakia mice with reduced PITX3 expression showed increased DA neuron loss in the SNpc after MPTP treatment. These results would suggest that PITX3 is neuroprotective, which is in contrast to the higher vulnerability of PITX3-positive SNpc neurons to MPTP than PITX3-negative neurons, and needs to be investigated in more detail to confirm that these observations are indeed due to the presence or absence of PITX3. Moreover, in these studies, MPTP was administered to mice at postnatal day 35, which represents a rather young mouse age at which DA neuron maturation may not entirely be completed, and thus the study results might be influenced by the contribution of developmental factors.

Another immunohistochemistry study in mouse and human tissue samples confirmed the DA neuron-specific expression of PITX3 in the midbrain (Reyes et al., 2013b). However, in humans, PITX3 was found to be expressed in more than 90% of DA neurons in both SNpc and VTA, suggesting that almost all DA neurons in both nuclei express some level of PITX3. In mice, more than 90% of SNpc DA neurons and about 80% of VTA DA neurons expressed PITX3, presenting a similar situation as in the human midbrain. The authors did not observe differences in the PITX3 expression pattern between the dorsal and ventral tiers of the SNpc. Interestingly, an analysis of publicly available human microarray data implied that PITX3 transcripts are enriched in the SNpc as compared to the VTA, possibly pointing to SNpc DA neurons expressing higher PITX3 levels than VTA DA neurons.

Taken together, various studies have described different expression patterns of PITX3 in the midbrain DA neurons even for the same species. The reason for these discrepancies is unknown, although it is possible that different degrees of sensitivity were reached with the different immunostaining protocols that were applied, resulting in the detection of low PITX3 levels in a subset of DA neurons in one study, while another study might have been less sensitive, leaving these DA neurons unlabeled. Therefore, it currently remains unclear if PITX3 can serve as a marker for subsets of midbrain DA neurons with different susceptibility to degeneration. Moreover, the data regarding the enrichment of PITX3 mRNA in the SNpc as compared to the VTA need to be supported by additional studies, as only a very limited number of subjects was investigated by Reyes et al. Furthermore, future investigations should focus on the function of PITX3 in adult DA neurons to identify the molecular pathways in which PITX3 is involved. These data may provide clues regarding the potential role of PITX3 in regulating the vulnerability of DA neurons to degeneration.

The nuclear receptor NURR1 is another transcription factor that is involved in the specification of midbrain DA neurons. *Nurr1* mRNA is detectable in the mouse midbrain as early as at embryonic day 10.5, and only after this time point, the expression of mRNAs encoding for DA neuron markers such as TH is induced (Zetterström et al., 1997). In *Nurr1* knockout mice, the final differentiation of DA precursors to neurons is inhibited and SNpc and VTA DA neurons are not generated in the midbrain (Zetterström et al., 1997; Castillo et al., 1998; Saucedo-Cardenas et al., 1998). Adult wild-type mice express NURR1 in almost all TH-positive neurons in the SNpc and the VTA, and several studies have demonstrated that NURR1 supports the maintenance and normal function of these neurons (Bäckman et al., 1999; Kadkhodaei et al., 2009, 2013). Differential expression of NURR1 in subgroups of midbrain DA neurons has not been observed, making it unlikely that NURR1 plays a role in selective DA vulnerability. Similarly, the homeobox proteins engrailed-1 and engrailed-2 are expressed in SNpc and VTA DA neurons in mice from early developmental stages into adulthood (Simon et al., 2001). Engrailed-1 seems to be expressed at high levels in all midbrain DA neurons, whereas engrailed-2 is mostly expressed at lower levels and high expression is found only in a subset of DA neurons. In particular, engrailed-1 is of paramount importance for the development of SNpc and VTA DA neurons, and also plays a role in their postnatal maintenance (Simon et al., 2001; Albéri et al., 2004; Sgadò et al., 2006; Sonnier et al., 2007; Nordström et al., 2014). However, based on the published data, the expression pattern of engrailed-1 does not differ between subgroups of DA neurons. Although it has been reported that the expression levels of engrailed-2 are not uniform among midbrain DA neurons in adult mice (Simon et al., 2001), a correlation between engrailed-2 levels and the differential susceptibility of DA neurons has not been carried out.

The neurogenesis of DA neurons in the mouse midbrain is partially controlled by the homeobox protein OTX2 (Omodei et al., 2008). Although OTX2 is expressed in all midbrain DA progenitors, this transcription factor appears to be of varying importance for the development of different subgroups of DA neurons (Di Giovannantonio et al., 2013). Based on data obtained in conditional *Otx2* knockout and *OTX2* overexpressing mice, it was suggested that OTX2 is of paramount importance for the generation of only a specific subgroup of VTA DA neurons (Di Giovannantonio et al., 2013). In adult mice, LCM of SNpc and VTA DA neurons combined with microarray expression analysis revealed that *Otx2* is about 6-fold enriched in the VTA as compared to the SNpc (Chung et al., 2005). This enrichment in the VTA was confirmed by the quantitative real-time PCR analysis of RNA samples collected from mouse or human SNpc and VTA DA neurons, respectively (Chung et al., 2010). Co-immunostaining of adult mouse midbrain for TH and OTX2 revealed OTX2 expression in many VTA DA neurons, but not in SNpc DA neurons (Chung et al., 2010; Di Salvio et al., 2010b). It was demonstrated that the DA neurons in the ventral and central VTA express OTX2. However, OTX2 expression was not exclusively restricted to DA neurons as some non-DA neurons in the VTA were also positive for OTX2 (Di Salvio et al., 2010b). Consistent with the data discussed above, it was observed that OTX2-expressing neurons are often positive for calbindin but only rarely co-express GIRK2. Moreover, the manipulation of OTX2 levels in midbrain DA neurons demonstrated that the depletion of OTX2 in the VTA leads to the expression of GIRK2, increased levels of the functional glycosylated form of DAT and increased neuronal vulnerability to MPTP, which is consistent with a potential neuroprotective role of OTX2 in VTA DA neurons (Di Salvio et al., 2010a). How OTX2 is able to control the expression of glycoslylated DAT and GIRK2 and the molecular pathway connecting altered DA neuron vulnerability with the expression of these proteins currently remains speculation. A neuroprotective role was also suggested by a different study in which OTX2 was either overexpressed or knocked down in primary mouse midbrain cultures using lentivirus technology (Chung et al., 2010). After MPP^+^ treatment, OTX2 overexpression resulted in an increased number of TH-positive neurons as compared to control cultures, whereas OTX2 knockdown led to a reduced number of TH-positive neurons, further indicating that OTX2 may decrease the susceptibility of DA neurons to toxin-induced degeneration.

Surprisingly, recent data presented by Reyes et al. imply that the potentially protective role of OTX2 in VTA DA neurons is not conserved between mice and humans (Reyes et al., 2013b). In agreement with previous analyses of adult mouse midbrain tissue, OTX2 immunoreactivity was detected in VTA DA neurons and not in SNpc DA neurons. These findings were consistent in tissue samples obtained from mice at different ages, ranging from four weeks to two years. In contrast, human SNpc and VTA DA neurons did not show any OTX2 expression either in middle-aged (58 ± 16 years old) or in aged control subjects (85 ± 3 years old) although a signal for OTX2 was detected in other brain regions. An analysis of *OTX2* mRNA levels in SNpc and VTA DA neurons in a 24-year-old and a 39-year-old individual suggested that *OTX2* expression is detectable at some point but severely declines with age. The latter data may also explain why Chung et al. were able to detect *OTX2* mRNA in laser-captured human VTA DA neurons (Chung et al., 2010). The findings by Reyes et al. would suggest that OTX2 does not play a neuroprotective role in aged human VTA DA neurons. However, these data need to be considered preliminary as only a very limited number of subjects were included in this analysis.

## Deleted in colorectal cancer

Besides transcription factors that play a role in the developing midbrain and that have been discussed in the previous section, another developmentally expressed protein has been investigated regarding its differential expression among adult midbrain DA neurons. Deleted in colorectal cancer (DCC), the receptor for the axon guidance molecule netrin, is located at the cell surface and highly expressed in the developing midbrain and at lower levels in the adult midbrain in rodents (Livesey and Hunt, 1997; Stein et al., 2001). The interaction between netrin and the DCC receptor is crucial for the guidance of SNpc and VTA DA axons to their respective striatal target regions (Li et al., 2014). Interestingly, in the adult rodent midbrain, co-immunostaining experiments revealed that DCC expression is largely restricted to TH-positive neurons, and most DCC-positive DA neurons were located in the ventral part of the SNpc (Osborne et al., 2005). Only few VTA DA neurons expressed DCC, and, for the most part, DCC-positive DA neurons in the SNpc and the VTA were negative for calbindin and vice versa. These data suggest that DCC may be useful as a marker for the population of SNpc DA neurons that are most vulnerable to degeneration. Reyes et al. investigated the DCC expression in human and mouse midbrain and observed that, in humans, more than 90% of DA neurons in the dorsal and ventral SNpc and in the VTA expressed this receptor. However, an overall enrichment of *DCC* mRNA in the SNpc as compared to the VTA was found, implying that DCC is expressed by almost all DA neurons, but the average *DCC* level may be higher in the DA neurons in the SNpc than in the VTA. In mouse midbrain, all ventral SNpc DA neurons expressed DCC, but only ~85% of dorsal SNpc neurons and ~62% of VTA DA neurons, somewhat resembling previously published data. Interestingly, in both human and mouse midbrain, ventral SNpc DA neurons showed a stronger signal for DCC than DA neurons in the dorsal SNpc and the VTA, suggesting that the DCC protein level may distinguish between populations of DA neurons with different vulnerability in both species. As with other investigated candidates, it remains unclear if the expression pattern of DCC is directly linked to the selective DA neuron vulnerability and which pathways DCC may use to modulate the susceptibility of DA neurons to degeneration.

## Mitochondria and energy demand

Several studies have investigated mitochondrial DNA deletions in SNpc DA neurons in human postmortem midbrain tissue samples. High levels of mitochondrial DNA deletions were found in SNpc DA neurons from both aged controls and patients with PD, while other brain regions were characterized by lower deletion levels (Bender et al., 2006, 2008; Kraytsberg et al., 2006). A recent study now compared mitochondrial DNA deletions in aged human control SNpc and VTA DA neurons using LCM followed by quantitative real-time PCR (Elstner et al., 2011). The number of detected deletions was higher in SNpc DA neurons than in VTA DA neurons, however, it is unclear if this difference is a cause or rather a consequence of the differential vulnerability observed among these two groups of neurons. Moreover, although noradrenergic neurons in the locus coeruleus are often more severely affected by degeneration in PD than SNpc DA neurons (Zarow et al., 2003), they showed fewer mitochondrial DNA deletions than SNpc and VTA DA neurons, suggesting that either the number of mitochondrial DNA deletions are unrelated to the selective vulnerability of DA and noradrenergic neurons, or the vulnerability of noradrenergic neurons is regulated by factors different from those that play a role for the vulnerability of SNpc and VTA.

Using immunostaining, Liang et al. studied the intracellular area that is occupied by mitochondria in various neurons in the mouse brain and used these data to draw conclusions regarding the mitochondrial mass (Liang et al., 2007). According to this investigation, SNpc DA neurons have a lower mitochondrial mass than VTA DA neurons, which might contribute to the selective vulnerability of SNpc and VTA. However, SNpc DA neurons appeared to have a similar mitochondrial mass as the much more resistant midline DA neurons in the interfascicular nucleus, and additional neuronal groups with a low mitochondrial mass were identified outside the midbrain which raises the question of how significantly the observed differences correlate with the selective vulnerability of these neurons to degeneration. Moreover, it is entirely unclear if these findings translate to the human brain.

Investigations addressing mitochondrial DNA deletions and the mitochondrial mass are interesting, particularly in the light of hypotheses arguing that the exceptionally high vulnerability of SNpc DA neurons as compared to VTA DA neurons may at least in part be due to their architecture and the resulting metabolic needs. SNpc DA neurons are estimated to form much larger axonal arbors and a higher number of synapses than VTA DA neurons, which may result in the pronounced redistribution of mitochondria to their axonal terminals and the tremendous elevation of their demand for energy as well as their susceptibility to insults jeopardizing the neuronal energy supply (Surmeier et al., 2010; Bolam and Pissadaki, 2012). Moreover, a recent computational analysis proposed that the unique architecture of SNpc DA neurons leads to a higher need of energy for the propagation of action potentials and the maintenance of the resting membrane potential (Pissadaki and Bolam, 2013). Closely related to this topic, a computational model for the degeneration of motor neurons in ALS predicted a link between mitochondrial dysfunction, the lack of ATP, prolonged depolarization and the disruption of intraneuronal calcium levels (Le Masson et al., 2014). Although very compelling, it needs to be pointed out that a significant part of these suggestions is based on computational evidence and not wet lab experiments. Actual comparative measurements of the energy demand of SNpc and VTA DA neurons, studies investigating and comparing the impact of axonal arborization on the distribution of mitochondria between terminals and neuronal cell bodies, and experiments providing direct evidence for a link between the lack of energy and high intracellular calcium levels have not been conducted to date.

## Aging

As aging is the major risk factor for developing PD, some investigations focus on the comparison of the changes that occur in SNpc and VTA DA neurons as a function of age, with the goal of identifying factors that mediate the selective vulnerability of midbrain DA neurons. Although interesting, this approach has been characterized by limited success to date. Using immunofluorescence staining of brain tissue obtained from young, middle-aged and old-aged rhesus monkeys, a recent study investigated the degree of nitrative damage in the midbrain DA neurons and concluded that DA neurons in the ventral SNpc show the most severe nitrative damage over time (Kanaan et al., 2008). Although interesting, these data are rather reflective of the differential DA vulnerability than providing an explanation for it. Gao et al. dissected and collected SNpc or VTA from mice of different ages and compared the molecular signature of the two brain regions using microarray (Gao et al., 2013). Differentially expressed genes were identified and grouped into functional categories. These results can serve as a database for follow-up studies that are required to confirm some of the differentially expressed gene candidates and to link their function to the selective vulnerability of different groups of DA neurons in the midbrain.

## Aldehyde dehydrogenase 1

Many studies have now provided evidence that molecular differences exist among SNpc DA neurons and among VTA DA neurons, and the most recent investigations addressing this topic have been discussed above. These data demonstrate that, within the SNpc and the VTA, subgroups of DA neurons exist that can be distinguished by subtle variations in their expression profiles. Such findings may potentially explain why not all DA neurons within the SNpc or the VTA demonstrate the same susceptibility to degeneration in PD. Furthermore, on the electrophysiologic level, Lammel et al. have shown that most VTA DA neurons are functionally different from SNpc DA neurons, and that considerable heterogeneity exists, especially among the DA neurons in the VTA (Lammel et al., 2008). However, an increasing body of evidence suggests that electrophysiologic heterogeneity can also be found among SNpc DA neurons. Activity recordings in monkeys demonstrated that functional diversity can be observed among SNpc DA neurons that project to different target regions, as not all of these neurons exhibit the same response to motivational stimuli (Matsumoto and Hikosaka, 2009). A different study in anesthetized mice revealed that burst firing *in vivo* is gated by ATP-sensitive potassium channels in some but not all SNpc DA neurons (Schiemann et al., 2012), and experiments carried out in mouse brain slices showed that some SNpc DA neurons are capable of co-releasing dopamine and GABA, but only a select subset of SNpc DA neurons has the capacity to do so (Tritsch et al., 2012). The molecular determinants of most of these differences and their potential relevance for the selective DA neuron vulnerability have not been identified to date. As discussed above, the higher vulnerability of ventral SNpc DA neurons as compared to dorsal SNpc DA neurons is one of the most well-known examples for heterogeneity among SNpc DA neurons. Interestingly, two studies in mice have demonstrated that aldehyde dehydrogenase 1 (ALDH1A1) is expressed in a subset of DA neurons, most of which are located in the ventrolateral part of the SNpc (McCaffery and Dräger, 1994; Liu et al., 2014). ALDH1A1 belongs to a group of enzymes that catalyze the oxidation of aldehydes to carboxylic acids. Aldehyde dehydrogenases such as ALDH1A1 presumably oxidize and thereby detoxify 3,4-dihydroxyphenylacetaldehyde (DOPAL), a reactive metabolite of dopamine (Marchitti et al., 2007). The inhibition of aldehyde dehydrogenase and the accumulation of DOPAL may contribute to the pathogenesis of PD (Fitzmaurice et al., 2013). Liu et al. now described a connection between the expression of ALDH1A1 and the vulnerability of DA neurons to alpha-synuclein-mediated degeneration. The authors focused their investigations on transgenic mice overexpressing human mutant alpha-synuclein in the midbrain DA neurons, resulting in moderate progressive DA neuron degeneration (Lin et al., 2012). Using these mice, it was shown that ALDH1A1-positive DA neurons in the ventral SNpc are more resistant to degeneration and develop fewer alpha-synuclein aggregates than ALDH1A1-negative SNpc DA neurons which are found in the dorsomedial part of the SNpc. Although very interesting, these findings are somewhat in contrast to the lower resistance of ventral tier SNpc DA neurons than dorsal tier SNpc DA neurons in PD (Kordower et al., 2013). ALDH1A1-positive VTA DA neurons also showed a higher resistance to degeneration than ALDH1A1-negative VTA DA neurons, indicating that ALDH1A1 is not a SNpc-specific susceptibility factor. Knockout of *Aldh1a1* alone was not sufficient to induce DA cell loss, but facilitated DA neuron degeneration in transgenic alpha-synuclein mice. Virus-mediated overexpression of ALDH1A1 in midbrain cultures from transgenic alpha-synuclein mice increased DA neuron survival. ALDH1A1-mediated protection seemed specific to the alpha-synuclein model and was not observed with the use of other neurotoxins *in vitro* which could point to a specific role for ALDH1A1 in diminishing alpha-synuclein-mediated toxicity. The exact mechanism, however, is unclear. The ALDH1A1 expression pattern in the midbrain was conserved between mice and humans. In midbrain tissue from human controls, ALDH1A1 expression was observed mainly in the ventral SNpc DA neurons. Tissue samples from PD patients revealed extensive DA cell loss in the ventral tier of the SNpc which seemed associated with the reduced expression of ALDH1A1 in the remaining ventral tier SNpc DA neurons at various disease stages. These data indicate that ALDH1A1 may potentially have a protective function for human SNpc DA neurons.

## Left-right-asymmetry

Last but not least, it is noteworthy that a number of studies discuss a differential vulnerability of SNpc DA neurons in the left and right hemispheres in PD. Many patients with PD demonstrate a pronounced asymmetric manifestation of the cardinal motor signs with one body side being more affected than the other side, and this asymmetry can also be measured in studies that image the DA system in PD patients using tracers (Djaldetti et al., 2006). These findings suggest that the left and right hemispheres contain a different number of SNpc DA neurons in these patients. A correlation of the number of neuromelanin-positive neurons in the SNpc bilaterally in post-mortem brain samples from PD patients with the asymmetric clinical signs that had been documented for these individuals revealed that many but not all cases showed the expected pattern of asymmetric cell loss (Kempster et al., 1989). Additional studies comparing the number of SNpc DA neurons in the left and right hemispheres in patients with PD have not been published. It is not well understood if the observed asymmetry is indeed due to the different vulnerability of left- and right-sided SNpc DA neurons to degeneration, or to other reasons including inborn, side-specific differences in the number of SNpc DA neurons, unbalanced limb exercise, hand dominance or the uneven, localized permeability of the blood brain barrier to toxins (Djaldetti et al., 2006). If differential susceptibility of the left and right SNpc to degeneration indeed plays a role, the molecular basis has yet to be elucidated. Interestingly, several asymmetric animal models have been developed recently, including a monkey model that was generated by a unilateral surgical lesion of the nigrostriatal pathway followed by systemic administration of MPTP, and a genetic DJ1 knockout mouse model (Blesa et al., 2011; Rousseaux et al., 2012). These models have the potential to serve as tools to further study the molecular basis of asymmetry in PD.

## Conclusion

The elucidation of the molecular determinants that increase the susceptibility of SNpc DA neurons or decrease the susceptibility of VTA DA neurons to degeneration in PD is a very exciting but challenging research area that holds great potential for the development of novel therapeutic strategies for PD. The gene expression signatures of SNpc and VTA DA neurons are extremely similar to each other. Only a rather limited number of genes within these two groups of neurons demonstrate differences in their expression levels, and the differences are usually small to moderate. Moreover, studies focusing on the identification and characterization of differentially expressed proteins have revealed that the candidate proteins identified to date are not exclusively expressed in either SNpc or VTA DA neurons but rather enriched in either neuronal group at best. Based on these findings, it may be speculated that it is unlikely that only one or two key proteins mediate the selective vulnerability of DA neurons in PD. Instead, it is more likely that a cell type-specific combination of several modest gene expression differences and subtle differences in protein function are responsible for establishing the increased or decreased midbrain DA neuron vulnerability. Although still at the beginning, current research on mitochondria and energy demand, the molecular underpinnings of electrophysiologic differences between subtypes of DA neurons, and developmentally expressed proteins that remain expressed in adult DA neurons appears promising. Emphasis should be placed on the identification of molecular targets that increase the resistance of DA neurons to degeneration. It is likely that multiple toxic factors contribute to DA neuron degeneration, and it will be difficult to slow down or halt cell loss by inhibiting only one or few of the related pathways. However, boosting the function of proteins that support the survival of SNpc DA neurons may provide a powerful tool to improve DA neuron resistance to many of the toxic pathways associated with PD. The non-uniform expression patterns of some proteins among SNpc as well as VTA DA neurons certainly add another layer of complexity to the investigation of differential DA neuron vulnerability. However, the careful consideration of these differences and the correlation of the expression pattern of these gene and protein candidates with the varying vulnerability of DA neuron subgroups may provide additional opportunities for the discovery of genes that play a role in the selective DA vulnerability in PD.

## Author contributions

Lars Brichta and Paul Greengard carried out the literature search, developed the concept for the review and wrote the manuscript.

## Conflict of interest statement

The authors declare that the research was conducted in the absence of any commercial or financial relationships that could be construed as a potential conflict of interest.
